# UglyTrees: a browser-based multispecies coalescent tree visualizer

**DOI:** 10.1093/bioinformatics/btaa679

**Published:** 2020-07-27

**Authors:** Jordan Douglas

**Affiliations:** School of Computer Science, University of Auckland, Auckland 1010, New Zealand

## Abstract

**Summary:**

Visualization is a vital task in phylogenetics and yet there is a deficit in programs which visualize the multispecies coalescent (MSC) model. UglyTrees (UT) is an easy-to-use program for visualizing multiple gene trees embedded within a single species trees. The mapping between gene and species nodes is automatically detected allowing for ready access to the program. UT can scrape the contents of a website for MSC analyses, enabling the sharing of interactive MSC figures through optional parameters in the URL. If a posterior distribution is uploaded, the transitions between MSC states are animated allowing the visual tracking of trees throughout the sequence.

**Availability and implementation:**

UT runs in all major web browsers including mobile devices, and is hosted at www.uglytrees.nz. The MIT-licensed code is available at https://github.com/UglyTrees/uglytrees.github.io.

## 1 Introduction

As biological sequence data become increasingly available, it becomes enticing to infer species phylogeny by concatenating genes sequences and inferring the phylogeny of the species as that of the gene tree. However, this approach makes for a biased estimator of species divergence times and substitution rates when incomplete lineage sorting is present (Arbogast *et al.*, 2002; Mendes and Hahn, 2016; Ogilvie *et al.*, 2016), and an inconsistent estimator of topology when divergence times are small (Pamilo and Nei, 1988). Bayesian multispecies coalescent (MSC) methods address these issues (Flouri *et al.*, 2018; Heled and Drummond, 2010; Höhna *et al.*, 2016; Jones, 2017; Ogilvie *et al.*, 2017; Ronquist *et al.*, 2012).

Visualization is an essential task in phylogenetics. Consequently, gene tree visualisation programs are ubiquitous [see Dendroscope—Huson *et al.* (2007); FigTree—Rambaut (2012); DensiTree—Bouckaert and Heled (2014); IcyTree—Vaughan (2017) and ape—Paradis and Schliep (2019)]. Unfortunately, MSC visualizers are far less common [the only program which we are aware of is a script used in Heled and Drummond (2010)].

In a conventional MSC depiction (Degnan and Rosenberg, 2009; Heled and Drummond, 2010; Rannala and Yang, 2003), one or more gene trees are embedded inside a species tree. Figure heights correspond to gene/species divergence times, while widths correspond to species’ (effective) population sizes.

In an MSC analysis, an arbitrary number of gene trees could be used, sometimes even hundreds or thousands (Ogilvie *et al.*, 2017; Singhal *et al.*, 2018). There is no guarantee that branches will not overlap. Moreover, although continuous population models exist (Heled and Drummond, 2010; Heled *et al.*, 2013), MSC analyses quite frequently invoke piecewise population size models where each species has its own freely determined population size (Gómez-Hernández *et al.*, 2019; Pinto *et al.*, 2019; Singhal *et al.*, 2018).

These two components of the MSC (embedded gene trees and piecewise population models) make its visualization an inherently inelegant task. This is compounded by the inverse relationship between the rate of coalescence and population size, which results in coalescent events tending to be clustered together in the narrowest of branches. This article presents UglyTrees (UT)—an easy-to-use browser-based program for visualizing MSC models. UT reads trees represented in Newick/NEXUS format and is therefore compatible with trees produced by *BEAST, StarBEAST2, STACEY, MrBayes and RevBayes (Heled and Drummond, 2010; Höhna *et al.*, 2016; Jones, 2017; Ogilvie *et al.*, 2017; Ronquist *et al.*, 2012).

## 2 Visualization of the MSC

UT renders zero-or-more (rooted binary) gene trees embedded within a single (rooted binary) species tree using scalable vector graphics (SVG). The tree parser is built on top of that of IcyTree (Vaughan, 2017). The mapping between gene and species nodes is automatically detected allowing for ready access to the program.

The mapping algorithm attempts to map each gene to exactly one species, first by direct substring comparison, and if that fails, the labels are split using a range of delimiters (‘_’, ‘-’ and ‘.’). If a mapping cannot be found, the user is prompted to give one. Consider the following example:

Genes: {horse_1, horse_2, seahorse_1}, Species: {horse, seahorse}. horse_1 and horse_2 are mapped to horse and seahorse_1 is mapped to seahorse.

**Fig. 1.**
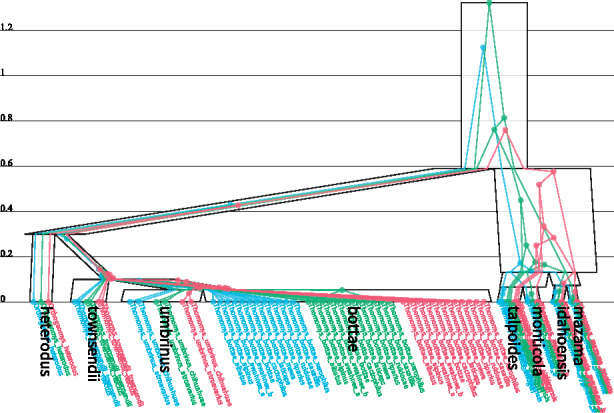
MSC analysis performed on three genes from a Gopher dataset (Belfiore *et al.*, 2008) using a piecewise constant population size model

The widths at the top and bottom of each species branch can be set independently (using tree meta-annotations) and the width in between is linearly interpolated (Fig. 1). This facilitates the visualization of two population size models commonly invoked in the literature: (i) piecewise *constant* models, for which each species branch has freely a determined population size (i.e. top and bottom are the same), and (ii) continuous *linear* models (Heled and Drummond, 2010), for which the population size at the bottom of each branch is equal to the sum of its two children’s population sizes at the tops of their respective branches.

If multiple MSC states are uploaded (a posterior distribution for instance), they can be iterated through with smooth animated transitions. This enables the visual tracking of trees through the posterior distribution. UT’s zooming feature makes it suitable for large datasets, however, performance depends on the number of SVG elements—with complexity dependent on the number of genes *G*, the number of species *S* and the taxon count *N*. When there is a large number of SVG elements, UT by default renders one gene tree at a time (Fig. 1).

## 3 Web scraping

Any changes made to the visual settings can be downloaded as a template in XML format. Display settings are restored upon subsequently uploading the template. By adding parameters to the URL, the simple backend of UT fetches a template file—and any tree files the template is pointing to—from the web. A customized message is optionally displayed to the user upon page load. This enables the sharing of MSC interactive visualizations with just one click. For example: http://uglytrees.nz/?w=http://uglytrees.nz/examples/gopher/session.xml

## Funding

This work was supported by Marsden grant [18-UOA-096], from the Royal Society of New Zealand.

*Conflict of Interest*: none declared.
